# Stress-Related Hair Loss Among the General Population in Al Majma'ah, Saudi Arabia: A Cross-Sectional Study

**DOI:** 10.7759/cureus.46517

**Published:** 2023-10-05

**Authors:** Abdulaziz S Alanazi, Waleed A Alsalhi, Waleed K Alghuyaythat, Abdulrahman N Almutairi, Mohanned A Almazrou, Jehad A Alabdulminaim, Elsadig Y Mohamed

**Affiliations:** 1 Department of Dermatology, King Khalid General Hospital, Hafar Al-Batin, SAU; 2 Department of Dermatology, College of Medicine, Majmaah University, Al Majma'ah, SAU; 3 College of Medicine, Majmaah University, Al Majma'ah, SAU; 4 Department of Community Medicine, College of Medicine, Majmaah University, Al Majma'ah, SAU

**Keywords:** majmaah, saudi arabia, psychosocial factors, sociodemographic factors, prevalence, hair loss, stress

## Abstract

Background

Hair loss is a prevalent concern affecting individuals worldwide, often attributed to various factors including genetics, hormonal changes, and stress. This study aims to investigate the prevalence of stress-related hair loss among the general population in Al Majma'ah, Saudi Arabia, and hair loss association with sociodemographic characteristics and risk factors.

Methods

A cross-sectional study was conducted involving 1080 participants. Data were collected through a structured questionnaire that encompassed sociodemographic factors, stress levels, psychosocial factors, and habits related to hair care and lifestyle. The data were analyzed using descriptive statistics, chi-square tests, and logistic regression analysis.

Results

The study revealed that 770 (71.3%) of participants reported experiencing hair loss, with females showing a higher prevalence compared to males 622 vs. 148 (78.2% vs. 51.9%). A significant relationship between age and hair loss was observed (Chi-x^2 = ^8.264, p-value = 0.016), with individuals aged 31 to 40 years experiencing hair loss more frequently 108 (80.6%). Stress was found to be a significant contributing factor to hair loss (χ^2 = 37.533, p < 0.001), with 674 (73.8%) of participants reporting stress. Moreover, stress levels exhibited a dose-response relationship with the severity of hair loss. Psychosocial factors, including personal relationship problems and financial difficulties, also demonstrated significant associations with hair loss (p-value = 0.005, 0.003, respectively).

Conclusion

The study underscores the considerable prevalence of stress-related hair loss among the general population in Al Majma'ah, Saudi Arabia. Stress, along with various sociodemographic and psychosocial factors, emerged as significant contributors to hair loss. These findings emphasize the need for holistic approaches that address both physiological and psychological aspects to mitigate the burden of hair loss in the community. Further research is warranted to explore the underlying mechanisms and develop targeted interventions for individuals at risk of stress-induced hair loss.

## Introduction

Normally, people have between 100,000 and 150,000 hairs on their heads. Hair has a programmed life cycle: a growth phase (anagen), a resting phase (catagen), and a shedding phase (telogen). The number of strands lost in a day varies but on average is 50-100. These strands lost usually don't cause noticeable hair loss because new hair is growing simultaneously. If this growth-loss cycle is disrupted, then hair loss (alopecia or baldness) is seen [1].

Hair loss is a common complaint in clinical practice. It can be classified as scaring and non-scaring. The causes of hair loss can fall into focal or diffuse alopecia. Focal hair loss may be caused by non-scaring and scaring alopecia. Non-scarring focal alopecia is usually caused by tinea capitis, alopecia areata, traction alopecia, or trichotillomania (where a person compulsively pulls out or breaks their hair). Scarring focal alopecia is rare and has several causes, usually discoid lupus erythematosus. The diffuse alopecia includes male and female hair patterns, diffuse alopecia areata, alopecia totalis or universals, and telogen effluvium. Telogen effluvium can be triggered by physiologic stress, such as surgical trauma, high fever, and chronic systemic illness. Emotional stress, medical conditions such as hypothyroidism or hyperthyroidism, and dietary deficiency can also cause diffuse telogen effluvium which can be reversible once the condition has been treated. In addition, multiple drugs can cause telogen effluvia like oral contraceptive pills, androgens, reti­noids, beta­-blockers, angiotensin-converting enzyme inhibitors, anti-convulsants, anti-depressants, and anti-coagulants [2-6].

In humans, hair loss is often reported clinically during periods of excessive stress, and especially when hair loss does not indicate underlying organic disease it can be disturbing for hair loss patients and their doctors [7,8]. It was therefore hypothesized that analogous to the animal models, in humans, stress activates neuroendocrine-immune circuits, which terminate hair growth in the absence of other clinically noticeable health disturbances [6,7]. Soon a person goes through a stressful event, hairs can go into the resting phase. Hair loss often becomes noticeable two-to-six months after the trauma.

As stress has long been considered as one of the main causal factors involved in hair loss, information about stress and hair loss interaction should be well known. With respect to the connections between psycho-emotional stress and hair loss, levels of interactions can be distinguished as acute or chronic stress as a primary inducer of telogen effluvium, acute or chronic stress as an aggravating factor in a hair loss disorder whose primary pathogenesis is of endocrine, toxic, metabolic, or immunological nature (e.g. AGA, alopecia areata) and stress as a secondary problem in response to prior hair loss. The latter might also contribute to the perpetuation or aggravation of hair loss and induce a self-perpetuating vicious circle [7].

Although there is a large body of research relating to stress and inflammation in animals and humans, which extensively examines cytokines and other pro-inflammatory effects of stress, only a small number of human studies have addressed the association between stress, immune response, and tissue regeneration in healthy, non-wounded humans, under prolonged, real life-stress exposure and as a result, no scientific evidence for stress-induced hair loss in humans is available to date [9-11].

Also, limited specific pharmacological intervention is currently available to manage stress-induced hair loss in men. An effective therapeutic intervention in this respect would have to prolong the anagen phase of the hair cycle, thus preventing the premature onset of catagen, which forms the basis of stress-induced telogen effluvium [12].

Limited research has been undertaken on the prevalence of hair loss and its relationship to stress in Al Majma'ah, KSA. This cross-sectional study will attempt to assess the prevalence of hair loss among the general population and to determine the relationship between stress and hair loss.

This study aims to determine the prevalence, causes of hair loss, and the relationship between stress and hair loss among the general population in Al Majma'ah, Saudi Arabia.

## Materials and methods

Study design

A descriptive cross-sectional community-based study was adopted to investigate the prevalence and factors associated with stress-related hair loss among the general population in Al Majma'ah, Saudi Arabia.

Participants

A randomized sample technique was recruited from the general population of Al Majma'ah. Inclusion criteria included being 18 years of age or older and willing to participate in the study. A sample size of 1080 participants was taken.

Data collection

Data were collected through an electronic pre-structured questionnaire, developed after expert consultation and extensive literature review. The questionnaire was designed in English language and translated to Arabic by an expert translator, and the data were collected in both Arabic and English language depending on what language the participant preferred. The questionnaire underwent validation by a panel of three experts to ensure its content validity and applicability. The reliability of the tool was assessed through a pilot study involving 15 participants, resulting in a reliability coefficient (α-Cronbach’s) of 0.71 for the satisfaction and perception section.

Questionnaire structure

The self-administered online questionnaire comprised two main sections. Section 1 gathered sociodemographic information, including age, sex, marital status, occupation, nationality, BMI group, and monthly income. Section 2 explored factors related to hair loss and stress, including questions about hair type, hair care practices, stress levels, psychosocial factors, and any history of depression or medication use.

Data collection process

The online questionnaire was distributed through popular social media platforms, including WhatsApp, Facebook, and Twitter. A brief introduction to the research's nature and the confidentiality of participants' information was provided to ensure ethical considerations.

Data analysis

Descriptive statistics, including frequencies and percentages, were employed to summarize the sociodemographic characteristics of the participants. The chi-square test was utilized to examine associations between sociodemographic factors and hair loss. Logistic regression analysis was employed to determine the relationship between stress and hair loss, adjusting for potential confounding variables.

Ethical considerations

The research proposal obtained approval from the Regional Research and Ethics Committee in Al Majma'ah. Permission was sought from the Joint Program of Family Medicine in Al Majma'ah, as well as the directors of the primary healthcare centers involved in the study.

## Results

In Table 1, we present the sociodemographic characteristics of the participants (n=1080) in the study on stress-related hair loss among the general population in Al Majma'ah, Saudi Arabia. The majority of participants were female, constituting 795 (73.6%) of the total sample, while males accounted for 285 (26.4%). Regarding age distribution, the highest proportion of participants fell within the 18 to 30 age group, making up 858 (79.4%) of the cohort. Participants aged 31 to 40 and 41 to 68 comprised 134 (12.4%) and 88 (8.1%), respectively.

**Table 1:** Hair loss and risk factors among participants (n=1080).

Parameter		Frequency (%)	Hair Loss		X^2	p-value
			Yes	No		
Sex	Female	795 (73.6%)	622 (78.2%)	173 (21.8%)	70.957	0.000
	Male	285 (26.4%)	148 (51.9%)	137 (48.1%)		
Age, y	18 to 30	858 (79.4%)	595 (69.3%)	263 (30.7%)	8.264	0.016
	31 to 40	134 (12.4%)	108 (80.6%)	26 (19.4%)		
	41 to 68	88 (8.1%)	67 (76.1%)	21 (23.9%)		
BMI group	Underweight (<18.5)	136 (12.6%)	96 (70.6%)	40 (29.4%)	4.318	0.229
	Normal (18.5 - 24.9)	584 (54.1%)	403 (69%)	181 (31%)		
	Overweight (25.0 -29.9)	222 (20.6%)	167 (75.2%)	55 (24.8%)		
	Obese (≥ 30.0)	138 (12.8%)	104 (75.4%)	34 (24.6%)		
Nationality	Non-Saudi	51 (4.7%)	35 (68.6%)	16 (31.4%)	0.186	0.666
	Saudi	1029 (95.3%)	735 (71.4%)	294 (28.6%)		
Marital status	Married	242 (22.4%)	188 (77.7%)	54 (22.3%)	13.977	0.001
	Divorced/Widowed	28 (2.6%)	26 (92.9%)	2 (7.1%)		
	Single	810 (75%)	556 (68.6%)	254 (31.4%)		
Educational status	Not studying	372 (34.4%)	285 (76.6%)	87 (23.4%)	11.053	0.001
	Still studying	708 (65.6%)	485 (68.5%)	223 (31.5%)		
Occupational status	Not working	554 (51.3%)	391 (70.6%)	163 (29.4%)	0.132	0.887
	Working	526 (48.7%)	379 (72.0%)	147 (28.0%)		
Hours of studying per day	I'm not studying	372 (34.4%)	285 (76.6%)	87 (23.4%)	12.269	0.015
	Less than 6 hours	231 (21.4%)	155 (67.1%)	76 (32.9%)		
	6 to 8 hours	357 (33.1%)	248 (69.5%)	109 (30.5%)		
	8 to 12 hours	87 (8.1%)	55 (63.2%)	32 (36.8%)		
	More than 12 hours	33 (3.1%)	27 (81.8%)	6 (18.2%)		
Hours of working per day	I'm not working	554 (51.3%)	391 (70.6%)	163 (29.4%)	3.094	0.542
	Less than 6 hours	142 (13.1%)	98 (69%)	44 (31%)		
	6 to 8 hours	243 (22.5%)	175 (72%)	68 (28%)		
	8 to 12 hours	115 (10.6%)	89 (77.4%)	26 (22.6%)		
	More than 12 hours	26 (2.4%)	17 (65.4%)	9 (34.6%)		
Monthly income per month, SAR	Less than 2000	613 (56.8%)	440 (71.8%)	173 (28.2%)	1.272	0.736
	2000 to 5000	190 (17.6%)	130 (68.4%)	60 (31.6%)		
	5000 to 10000	130 (12%)	96 (73.8%)	34 (26.2%)		
	More than 10000	147 (13.6%)	104 (70.7%)	43 (29.3%)		
Hours of leisure activity per day	Less than 6 hours	853 (79%)	621 (72.8%)	232 (27.2%)	6.338	0.096
	6 to 8 hours	175 (16.2%)	118 (67.4%)	57 (32.6%)		
	8 to 12 hours	28 (2.6%)	18 (64.3%)	10 (35.7%)		
	More than 12 hours	24 (2.2%)	13 (54.2%)	11 (45.8%)		
Hours of physical activity per day	Less than 6 hours	880 (81.5%)	629 (71.5%)	251 (28.5%)	8.350	0.039
	6 to 8 hours	139 (12.9%)	102 (73.4%)	37 (26.6%)		
	8 to 12 hours	42 (3.9%)	31 (73.8%)	11 (26.2%)		
	More than 12 hours	19 (1.8%)	8 (42.1%)	11 (57.9%)		
Do you follow a diet (contains fruit, vegetables, and proteins)?	Yes	370 (34.3%)	248 (67%)	122 (33%)	5.013	0.025
	No	710 (65.7%)	522 (73.5%)	188 (26.5%)		
Cups of coffee/tea per day	None	20 (1.9%)	12 (60%)	8 (40%)	2.514	0.642
	More than three	221 (20.5%)	159 (71.9%)	62 (28.1%)		
	One	356 (33%)	248 (69.7%)	108 (30.3%)		
	Three	167 (15.5%)	124 (74.3%)	43 (25.7%)		
	Two	316 (29.3%)	227 (71.8%)	89 (28.2%)		
Smoking	Yes	126 (11.7%)	78 (61.9%)	48 (38.1%)	6.148	0.013
	No	954 (88.3%)	692 (72.5%)	262 (27.5%)		
Family history of hair loss	Yes	483 (44.7%)	392 (81.2%)	91 (18.8%)	41.536	0.000
	No	597 (55.3%)	378 (63.3%)	219 (36.7%)		
Total		1080	310( 28.7%)	770 (71.3%)	-	-

BMI groups were also analyzed, revealing that the largest proportion of participants fell within the "Normal" BMI category (18.5 to <25.0), comprising 584(54.1%) of the sample. Other BMI categories included "Underweight" - BMI <18.5 - (136, 12.6%), "Overweight" - BMI 25.0 to < 30.0 - (222, 20.6%), and "Obese" - BMI ≥ 30.0 - (138, 12.8%).

In terms of nationality, the overwhelming majority of participants were Saudi, accounting for 1029 (95.3%) of the total, while non-Saudi participants constituted 51 (4.7%). Marital status distribution showed that the majority of participants were single 810 (75%), followed by married individuals 242 (22.4%), and a smaller proportion of divorced/widowed participants 28 (2.6%).

Regarding educational status, the majority of participants were currently studying (708, 65.6%), with 373(34.4%) not pursuing studies. Occupational status indicated that more than half of the participants were not working (554, 51.3%), while 526 (48.7%) were employed.

Participants' daily study hours revealed that a substantial number reported not studying 372 (34.4%), while the highest proportion reported studying for 6 to 8 hours per day 357 (33.1%). When it came to daily working hours, a significant proportion were not working 554 (51.3%), while 243 (22.5%) reported working 6 to 8 hours per day.

Monthly income distribution indicated that the largest proportion (613 (56.8%)) of participants had an income of less than 2000 Saudi Riyals, while 190 (17.6%) had an income between 2000 and 5000 Saudi Riyals, 130 (12%) between 5000 and 10000 Saudi Riyals, and 147 (13.6%) reported an income higher than 10000 Saudi Riyals.

Leisure activities and physical activity patterns were also explored. The majority of participants reported engaging in leisure activities for less than six hours per day 853 (79%), and physical activity for less than six hours per day 880 (81.5%).

When it came to dietary habits, 710 (65.7%) of participants reported not following a diet containing fruit, vegetables, and proteins, while 370 (34.3%) indicated that they did follow such a diet.

Participants' consumption of coffee and tea was also assessed. The majority reported consuming one cup per day (356 participants (33%)), followed by two cups by 316 participants (29.3%) and three cups by 167 participants (15.5%). A smaller percentage reported consuming no coffee or tea 20 (1.9%), while 221 (20.5%) consumed more than three cups per day.

Smoking habits were analyzed, with the majority of participants indicating that they did not smoke954 (88.3%), while 126 (11.7%) reported being smokers. Additionally, family history of hair loss was explored, revealing that 483 (44.7%) of participants reported having a family history of hair loss, while 597 (55.3%) did not have such a history.

Table 1 presents the relationship between hair loss and various sociodemographic characteristics among the study participants. The analysis revealed several noteworthy patterns. Firstly, a statistically significant association was observed between sex and hair loss (χ^2 = 70.957, p < 0.001). Females exhibited a higher prevalence of hair loss 622 (78.2%) compared to males 148 (51.9%). Age also demonstrated a significant correlation with hair loss (χ^2 = 8.264, p = 0.016). Participants aged 31 to 40 years had the highest proportion of hair loss vs no hair loss, while the proportion decreased among those aged 41 to 68 and 18 to 30 (80.6% vs 76.1% vs 69.3%). Regarding marital status, a significant relationship was found (χ^2 = 13.476, p = 0.001), with divorced or widowed individuals showing a higher prevalence of hair loss 26 (92.9%). Interestingly, participants who reported not studying exhibited a higher prevalence of hair loss (285, 76.6%) compared to those still studying (485, 68.5%), demonstrating a statistically significant association (χ^2 = 11.053, p = 0.001).

Furthermore, participants who reported not working had a slightly higher prevalence of hair loss (391, 72.0%) compared to those who were working (379, 70.6%), although this association was not statistically significant (χ^2 = 0.132, p = 0.887). Notably, the presence of a family history of hair loss exhibited a strong correlation with hair loss (χ^2 = 41.466, p < 0.001), with individuals having a family history of hair loss showing a higher prevalence 392 (81.2%) compared to those without such a history378 (63.3%). 

As seen in Table 2, the hair type was examined in relation to hair loss, revealing that participants with different hair types reported experiencing hair loss at varying frequencies. Those with "Heavy curled" hair type comprised a smaller proportion (56, 5.2%) of the sample, while "Curled" (478, 44.3%) and "Straight" (546, 50.6%) hair types were more prevalent.

**Table 2:** Hair loss in association with risk factors among participants (n=1080).

Parameter		Frequency (%)	Hair Loss		X^2	p-value
			Yes	No		
Your actual hair type	Curled	478 (44.3%)	340 (71.1%)	138 (28.9%)	7.128	0.068
	Heavy curled	56 (5.2%)	38 (67.9%)	18 (32.1%)		
	Straight	546 (50.6%)	392 (71.8%)	154 (28.2%)		
Number of times of using heat to straight hair per week	None	526 (48.7%)	358 (68.1%)	168 (31.9%)	6.772	0.148
	One time	222 (20.6%)	171 (77%)	51 (23%)		
	Two times	144 (13.3%)	106 (73.6%)	38 (26.4%)		
	Three times	96 (8.9%)	70 (72.9%)	26 (27.1%)		
	More than three times	92 (8.5%)	65 (70.7%)	27 (29.3%)		
Number of times the hair combed per week	None	98 (9.1%)	64 (65.3%)	34 (34.7%)	9.121	0.104
	One time	59 (5.5%)	36 (61%)	23 (39%)		
	Two times	98 (9.1%)	73 (74.5%)	25 (25.5%)		
	Three times	131 (12.1%)	96 (73.3%)	35 (26.7%)		
	More than three times	151 (14%)	118 (78.1%)	33 (21.9%)		
	Every day	543 (50.3%)	383 (70.5%)	160 (29.5%)		
Number of shampooing per week	None	12 (1.1%)	6 (50%)	6 (50%)	21.498	0.001
	One time	59 (5.5%)	34 (57.6%)	25 (42.4%)		
	Two times	167 (15.5%)	127 (76%)	40 (24%)		
	Three times	374 (34.6%)	274 (73.3%)	100 (26.7%)		
	More than three times	249 (23.1%)	191 (76.7%)	58 (23.3%)		
	Every day	219 (20.3%)	138 (63%)	81 (37%)		
Regular coloring	Yes	235 (21.8%)	175 (74.5%)	60 (25.5%)	1.477	0.224
	No	845 (78.2%)	595 (70.4%)	250 (29.6%)		
Presence of pathological condition associated with hair loss	Yes	150 (13.9%)	131 (87.3%)	19 (12.7%)	21.891	0.000
	No	930 (86.1%)	639 (68.7%)	291 (31.3%)		
Do you suffer from stress?	Yes	913 (84.5%)	674 (73.8%)	239 (26.2%)	37.533	0.000
	No	167 (15.5%)	96 (57.5%)	71 (42.5%)		
Level of stress	I don't have	167 (15.5%)	96 (57.5%)	71 (42.5%)	41.034	0.000
	Low	212 (19.6%)	134 (63.2%)	78 (36.8%)		
	Moderate	464 (43%)	342 (73.7%)	122 (26.3%)		
	Severe	237 (21.9%)	198 (83.5%)	39 (16.5%)		
Do you have personal relationship problems?	Yes	339 (31.4%)	261 (77%)	78 (23%)	7.830	0.005
	No	741 (68.6%)	509 (68.7%)	232 (31.3%)		
Do you have problems with work or studying?	Yes	358 (33.1%)	266 (74.3%)	92 (25.7%)	2.364	0.124
	No	722 (66.9%)	504 (69.8%)	218 (30.2%)		
Do you have financial problems?	Yes	344 (31.9%)	266 (77.3%)	78 (22.7%)	8.967	0.003
	No	736 (68.1%)	504 (68.5%)	232 (31.5%)		
Felt that things were going your way?	Always	92 (8.5%)	65 (70.7%)	27 (29.3%)	41.907	0.000
	Sometimes	766 (70.9%)	585 (76.4%)	181 (23.6%)		
	Never	222 (20.6%)	120 (54.1%)	102 (45.9%)		
Found that you could NOT cope with all the things you had to do?	Always	93 (8.6%)	75 (80.6%)	18 (19.4%)	27.096	0.000
	Sometimes	696 (64.4%)	521 (74.9%)	175 (25.1%)		
	Never	291 (26.9%)	174 (59.8%)	117 (40.2%)		
Felt difficulties were piling up so high that you could not overcome them?	Always	167 (15.5%)	134 (80.2%)	33 (19.8%)	41.907	0.000
	Sometimes	663 (61.4%)	488 (73.6%)	175 (26.4%)		
	Never	250 (23.1%)	148 (59.2%)	102 (40.8%)		
Felt that you were unable to control important things in your life?	Always	133 (12.3%)	112 (84.2%)	21 (15.8%)	35.020	0.000
	Sometimes	627 (58.1%)	467 (74.5%)	160 (25.5%)		
	Never	320 (29.6%)	191 (59.7%)	129 (40.3%)		
Do you have any depression signs (change in appetite, suicidal idea, sleep disturbance, loss of interest, feeling depressed, etc.)?	Yes	523 (48.4%)	398 (76.1%)	125 (23.9%)	11.432	0.001
	No	557 (51.6%)	372 (66.8%)	185 (33.2%)		
Do you suffer from hair loss more during stress periods?	Yes	558 (51.7%)	476 (85.3%)	82 (14.7%)	110.702	0.000
	No	522 (48.3%)	294 (56.3%)	228 (43.7%)		
Do you use any antidepressant?	Yes	65 (6%)	45 (69.2%)	20 (30.8%)	0.144	0.704
	No	1015 (94%)	725 (71.4%)	290 (28.6%)		
	Do you use any drugs associated with hair loss? (e.g.: androgens, beta-blockers, antic-convulsants, anti-depressants, anti-coagulants, etc.)	Yes	141 (13.1%)	118 (83.7%)			23 (16.3%)	12.168	0.000
No		939 (86.9%)	652 (69.4%)	287 (30.6%)					
Did you feel that you lost your hair when you were been pregnant?	I have never been pregnant before/I'm male	746 (69.1%)	519 (69.6%)	227 (30.4%)	-	-
	Yes	101 (9.4%)	86 (85.1%)	15 (14.9%)	11.641	0.003
	No	233 (21.6%)	165 (70.8%)	68 (29.2%)		
Total		1080	310 (28.7%)	770 (71.3%)	-	-

The frequency of using heat to straighten hair per week was explored. Participants using heat "more than three times" 96 (8.5%) and "one time" 222 (20.6%) reported varying levels of hair loss, while those who did not use heat accounted for 526 (48.7%) of the sample.

Combing habits were investigated, with "every day" combing reported by 543 (50.3%) of participants. The relationship between combing frequency and hair loss was notable, as participants who combed "more than three times" 151 (14%) and "three times" 131 (12.1%) also reported experiencing hair loss.

The frequency of shampooing per week was examined, with participants reporting varying levels of hair loss based on shampooing habits. Those who shampooed "every day" 219 (20.3%) and "more than three times" 249 (23.1%) showed different patterns of hair loss.

The impact of regular coloring on hair loss was assessed, with participants who reported "No" regular coloring accounting for 845 (78.2%) of the sample, and those reporting "Yes" comprising 235 (21.8%).

The relationship between self-reported hair loss and other factors such as the presence of a pathological condition associated with hair loss, stress levels, personal relationships, work or studying issues, and financial problems was analyzed. Interestingly, participants who reported suffering from hair loss during the stress period (558 (51.7%)) were almost equal to those who did not (522 (48.3%)). The participants' stress levels were further examined, revealing that varying levels of stress were associated with different proportions of hair loss. Those reporting "Moderate" stress (464 (43%)) and "Severe" stress (237 (21.9%)) showed different patterns of hair loss.

Moreover, the presence of depression signs was assessed, demonstrating a connection between depression and hair loss, as participants reporting "Yes" to depression signs accounted for 523(48.4%) of the sample, and 398(76.1%) of them suffered from hair loss.

The potential influence of pregnancy on hair loss was explored. Participants who reported having "never been pregnant before/I'm male" 746 (69.1%), "No" 233 (21.6%), and "Yes" 101 (9.4%) showed varying patterns of hair loss during pregnancy. Furthermore, the use of antidepressants and drugs associated with hair loss was investigated, indicating that participants who reported using these medications accounted for 56 (6%) and 141 (13.1%) of the sample, respectively.

Table 2 explores the relationship between hair loss and various risk factors and psychosocial variables. Notably, a statistically significant association was observed between stress and hair loss (χ^2 = 37.533, p < 0.001). Participants who reported suffering from stress had a notably higher prevalence of hair loss 674 (73.8%) compared to those who did not report stress 96 (57.5%). This finding underscores the potential influence of stress on hair loss. Additionally, the severity of stress demonstrated a statistically significant association with the severity of hair loss (χ^2 = 41.034, p < 0.001).

A logistic regression was performed to ascertain the effect of stress and level of stress on the likelihood that participants have hair loss. The logistic regression model was statistically significant X2 (2) = 48.917, p < 0.001. The model explained 33.0% (Nagelkerke R2) of the variance in heart disease and correctly classified 71.0% of cases. Participants who suffered from stress were 3.04 times (95% C.I.) more likely to exhibit hair loss than participants who didn’t suffer from stress. Increasing stress level severity was associated with an increased likelihood of exhibiting hair loss, with 5.64 times (95% C.I.) more likely to exhibit hair loss in participants who had severe stress compared to participants who didn’t suffer from stress (data not shown).

Moreover, several psychosocial factors exhibited significant associations with hair loss. Participants who reported having personal relationship problems were more likely to experience hair loss (261 (77%)) compared to those without such problems 232 (31.3%), revealing a significant correlation (χ^2 = 7.830, p = 0.005). Similarly, individuals who reported facing challenges with work or studying had a higher prevalence of hair loss (266 (74.3%)) compared to those without such challenges (218 (30.2%)), although this association was not statistically significant (χ^2 = 2.364, p = 0.124). A particularly strong association was observed between feeling unable to cope with tasks and hair loss (χ^2 = 27.096, p < 0.001), as well as between feeling unable to control important aspects of life and hair loss (χ^2 = 35.020, p < 0.001). Participants who reported having financial problems were more likely to experience hair loss 266 (77.3%) compared to those without financial problems (504 (68.1%)), showing a statistically significant association (χ^2 = 8.967, p = 0.003). Finally, participants who reported experiencing depression signs had a higher prevalence of hair loss (398 (76.1%)) compared to those without such signs (185 (33.2%)), showing a statistically significant relationship (χ^2 = 11.432, p = 0.001).

## Discussion

The present study aimed to explore the prevalence of stress-related hair loss and its relationship with various sociodemographic characteristics and risk factors among the general population in Al Majma'ah, Saudi Arabia. The results provided significant insights into the intricate interplay between stress, sociodemographic factors, and the occurrence of hair loss.

The findings indicated a considerable prevalence of hair loss among the study population, with 770 (71.3%) of participants reporting experiencing hair loss. This prevalence aligns with existing literature, which suggests that hair loss is a common phenomenon that affects a substantial proportion of the global population regardless of age, sex, and ethnicity [13]. In line with prior research, females reported a higher frequency of hair loss than males [6,13]. Furthermore, age emerged as a noteworthy factor, with participants aged 31 to 40 (80.6%) experiencing hair loss more frequently than other age groups. This observation resonates with studies that associate age with increased vulnerability to hair loss due to physiological changes and hormonal shifts [13,14].

The investigation into the relationship between stress and hair loss yielded compelling results. A significant association was established between stress and hair loss, with 674 (73.8%) of participants reporting suffering from stress. This aligns with a body of literature that underscores the connection between chronic stress and hair loss [13,15]. Moreover, the level of stress exhibited a significant relationship with hair loss, with higher stress levels correlating with an elevated prevalence of hair loss. This finding corroborates existing research suggesting that increased stress levels can trigger or exacerbate hair loss [15].

The study also delved into psychosocial factors contributing to hair loss. Personal relationship problems, work/study-related challenges, and financial difficulties exhibited significant associations with hair loss. These findings are consistent with studies emphasizing the role of psychosocial stressors in contributing to hair loss [11,16]. The substantial impact of such factors underscores the importance of addressing holistic well-being in hair loss prevention and management strategies.

Comparing the current study's findings with existing literature reveals both concurrences and disparities. While the prevalence of hair loss aligns with previous research, the influence of specific sociodemographic factors on hair loss seems to exhibit variability across populations, such as in age, gender, and BMI [13,16]. The significance of stress as a contributing factor is a consistent theme, emphasizing its universal impact on hair health [11,13]. However, the association between stress levels and the severity of hair loss necessitates further exploration to better understand the underlying mechanisms [11].

This study's findings hold implications for both clinical practice and public health initiatives. The comprehensive understanding of the interplay between stress, sociodemographic factors, and hair loss can guide healthcare professionals in developing personalized interventions. Public health campaigns targeting stress management and psychosocial well-being could potentially mitigate the burden of hair loss in the community.

Future research endeavors should delve deeper into the underlying physiological mechanisms linking stress with hair loss. Additionally, longitudinal studies could provide insights into the temporal dynamics of stress-induced hair loss and identify potential intervention windows. Exploring the impact of cultural norms and lifestyle factors specific to the Saudi Arabian context on hair health could further enrich our understanding.

Limitations of the current study is represented by the descriptive cross-sectional design that limits its evidence strength. Furthermore, as a questionnaire-based study, it is subjected to a recall bias in which participants are largely dependent on pure memory to answer the questions. Finally, as the study was conducted through an online survey only, the findings represented the study sample only and cannot be generalized. 

## Conclusions

In conclusion, this study significantly contributes to the growing body of knowledge on stress-related hair loss by shedding light on the prevalence, associated risk factors, and their interactions among the general population in Al Majma'ah, Saudi Arabia. The findings highlight the clear interplay between stress, sociodemographic characteristics, and hair loss. By advancing our comprehension of these relationships, this research paves the way for more targeted interventions to address hair loss and enhance overall well-being.
